# Results of a pilot course to introduce palliative care to ICU nurses

**DOI:** 10.1186/2197-425X-3-S1-A715

**Published:** 2015-10-01

**Authors:** F DeKeyser Ganz, M Ben Nun, O Raanan

**Affiliations:** Hadassah Hebrew University, School of Nursing, Jerusalem, Israel; Belinson Medical Center, General Intensive Care Unit, Petach Tikva, Israel; Sheba Medical Center, School of Nursing, Tel Hashomer, Israel

## Introduction

While palliative care has been practiced for several decades, its principles are just beginning to be actively implemented in many ICUs across Europe. The Israeli Society for Cardiac and Intensive Care Nursing developed and administered an innovative ICU Palliative Care course. The primary goal of this course was not only to educate those nurses that participated in the course, but to change End-of-Life Palliative Care practices, leading to improved quality of care. A study was conducted to determine whether the course influenced participants with regards to their knowledge, attitudes, behaviors and end-of-life clinical practices.

## Objectives

To determine the effect of the ICU Palliative Care course on self-perceived knowledge, attitudes, and behaviors; and end-of-life clinical practices.

## Methods

34 ICU nurse participants of the course completed 4 questionnaires (a personal characteristics questionnaire; End-of-Life Care in the ICU; Quality of Death and Dying; End-of-Life Nursing Practices) and participated in a “Knowledge Café” on the first and last days of the course, 6 months apart.

## Results

Participants reported high levels of satisfaction with the course. Palliative Care knowledge, attitude and behaviors were significantly improved during the 6 months of the course (see table). The Knowledge Cafe found that participants expressed more willingness to provide PC to their patients, increased sensitivity to the holistic needs of patients and families, and increased confidence in the initiation of end-of-life discussions with their colleagues. Table 1.

**Table 1:** Table 1

Knowledge:	T1:4.1+.63	T2: 4.5+.41	p=.023
Attitudes	T1: 3.9+.70	T2: 4.3+.57	p=.01
Behaviors	T1: 3.0+ .75	T2: 3.4+ .78	p=.03
Quality of Death and Dying:	T: 6.97+ 2.2	T2: 7.04+1.9	p=.37

## Conclusions

The course achieved its goal of educating ICU nurses about Palliative Care and has started to trigger improved ICU Palliative Care practices. It is recommended that similar courses be developed and implemented in other countries across Europe.

